# A method for evaluation of patient-specific lean body mass from limited-coverage CT images and its application in PERCIST: comparison with predictive equation

**DOI:** 10.1186/s40658-021-00358-7

**Published:** 2021-02-08

**Authors:** Jingjie Shang, Zhiqiang Tan, Yong Cheng, Yongjin Tang, Bin Guo, Jian Gong, Xueying Ling, Lu Wang, Hao Xu

**Affiliations:** Department of Nuclear Medicine and PET/CT-MRI Center, The First Affiliated Hospital of Jinan University, No. 613 West Huangpu Road, Guangzhou, 510630 China

**Keywords:** Lean body mass, PERCIST 1.0, Response evaluation

## Abstract

**Background:**

Standardized uptake value (SUV) normalized by lean body mass ([LBM] SUL) is recommended as metric by PERCIST 1.0. The James predictive equation (PE) is a frequently used formula for LBM estimation, but may cause substantial error for an individual. The purpose of this study was to introduce a novel and reliable method for estimating LBM by limited-coverage (LC) CT images from PET/CT examinations and test its validity, then to analyse whether SUV normalised by LC-based LBM could change the PERCIST 1.0 response classifications, based on LBM estimated by the James PE.

**Methods:**

First, 199 patients who received whole-body PET/CT examinations were retrospectively retrieved. A patient-specific LBM equation was developed based on the relationship between LC fat volumes (FV_LC_) and whole-body fat mass (FM_WB_). This equation was cross-validated with an independent sample of 97 patients who also received whole-body PET/CT examinations. Its results were compared with the measurement of LBM from whole-body CT (reference standard) and the results of the James PE. Then, 241 patients with solid tumours who underwent PET/CT examinations before and after treatment were retrospectively retrieved. The treatment responses were evaluated according to the PE-based and LC-based PERCIST 1.0. Concordance between them was assessed using Cohen’s κ coefficient and Wilcoxon’s signed-ranks test. The impact of differing LBM algorithms on PERCIST 1.0 classification was evaluated.

**Results:**

The FV_LC_ were significantly correlated with the FM_WB_ (*r*=0.977). Furthermore, the results of LBM measurement evaluated with LC images were much closer to the reference standard than those obtained by the James PE. The PE-based and LC-based PERCIST 1.0 classifications were discordant in 27 patients (11.2%; *κ* = 0.823, *P*=0.837). These discordant patients’ percentage changes of peak SUL (SUL_peak_) were all in the interval above or below 10% from the threshold (±30%), accounting for 43.5% (27/62) of total patients in this region. The degree of variability is related to changes in LBM before and after treatment.

**Conclusions:**

LBM algorithm-dependent variability in PERCIST 1.0 classification is a notable issue. SUV normalised by LC-based LBM could change PERCIST 1.0 response classifications based on LBM estimated by the James PE, especially for patients with a percentage variation of SUL_peak_ close to the threshold.

## Background

Quantitative ^18^F-fluorodeoxyglucose positron emission tomography (^18^F-FDG PET) is expected to play a major role in assessing whether a tumour is responding to therapy, allowing physicians then to quickly determine whether to continue, change, or abandon treatment [1–3]. Fundamentally, quantitative PET treatment response assessment is based on the alteration in the standardized uptake value (SUV) between baseline and follow-up studies. But many technical factors affect SUVs and may lead to spurious variation [4]. To ensure the accuracy of assessment results, the PET evaluation response criteria in solid tumours (PERCIST) 1.0 have been proposed to mitigate these problems [5].

Nevertheless, there are still many sources of error in SUV measurement [6]. PERCIST 1.0 recommends using lean body mass (LBM) normalised SUV (SUL), due to its less variable between individuals of different body weights [5]. Modern PET/CT scanners commonly use the James predictive equation (PE), which relies on sex, height (cm), and total body weight (kg), to estimate LBM [7]. However, this equation has some limitations that weaken its reliability. A previous study has shown that the James PE was significantly different from LBM determined from dual-energy X-ray absorptiometry, which is one of the best reference methods [8]. In addition, Tahari et al. found inappropriately low hepatic SUL values in the very obese female patients when using this PE [9]. Therefore, a more accurate method should be proposed to ensure the reliability of LBM.

Computed tomography (CT) has become a standard method for measuring body composition [10]. Because different tissue types vary in X-ray attenuation on the basis of their different densities and chemical compositions, they can be distinguished on CT images. Thus, LBM can also be measured from the CT image obtained during the PET/CT examination [11]. However, conventional PET/CT examinations for most situations covered only the range from skull vertex to upper thighs and could not measure whole-body LBM. With this in mind, some have proposed using the limited-coverage (LC) CT to estimate LBM and demonstrated that LBM estimated from LC images has an excellent agreement with LBM measured on a whole-body CT [12, 13].

Intuitively, more accurate measurement of LBM estimates from LC images should produce more accurate SUL normalisations than LBM estimates from the James PE. Narita et al. [14] reported that, compared with James PE-based SUL, individual CT-derived SUL could provide more stable hepatic uptake values that are available for PERCIST 1.0. However, the impact of the LBM algorithm on the PERCIST 1.0 classification of treatment response is unclear. PERCIST 1.0 suggests a threshold of 30% change in SUL (combined with a minimal absolute change) to define either partial response or progressive disease [5]. In practice, whether the difference between SUL normalisations would influence the response classifications, and how much the influence would be, has not been reported. Therefore, the aims of this study are to introduce a novel and reliable method to estimate LBM by LC images from PET/CT examinations and compare the results with LBM estimates of the James PE. Then, analysis of whether SUV normalised by LC images-based LBM could change the PERCIST 1.0 response classifications based on LBM estimated by the James PE.

## Methods

### Patients

First, a total of 296 patients who received whole-body ^18^F-FDG PET/CT examinations between December 2011 and March 2018 were retrospectively reviewed. All patients were randomly assigned into equation-development (106 men and 93 women) and validation (54 men and 43 women) groups. Then, patients with solid tumours who underwent ^18^F-FDG PET/CT before and after treatment were retrospectively retrieved as the application group between the same periods. The inclusion criteria of this group were as follows: non-diabetic, still-existing ^18^F-FDG-avid lesions and no appearance of new ^18^F-FDG-avid lesions after treatment. Finally, a total of 241 patients (145 men and 96 women) were included in this study, including 72 with non–small cell lung cancer, 53 with hand and neck tumours, 46 with breast cancer, 22 with oesophageal cancer, and 48 with colorectal cancer. The clinic variables of patients were recorded, which included sex, age, height, weight, body-mass index (BMI), blood glucose level, injected dose of ^18^F-FDG, and uptake time. The study was approved by the institutional review board of the First Affiliated Hospital of Jinan University and complied with national legislation and the Declaration of Helsinki guidelines. All patients gave consent to use their ^18^F-FDG PET/CT results and relevant clinical data for this study.

### PET/CT examinations

All ^18^F-FDG PET/CT examinations were performed with the same protocol using a GE Discovery PET/CT 690 system. PET/CT images were acquired 50 to 70 min after intravenous injection of ^18^F-FDG at a dose of 0.08–0.10 mCi/kg body weight. The scanning range covered the whole-body for the equation-development and validation groups and covered from the top of the skull to mid-thigh for the application group. CT data were acquired in breath-hold with 120 kV, 80–160 mA modulated using the GE AutomA technique with a slice thickness of 3.75 mm, slice interval of 3.27 mm, pitch of 1.375, matrix size of 512 × 512 and scan field-of-view of 50 cm. PET data were acquired in 3D time-of-flight (TOF) mode with a 2-min scan per bed position, overlap of 23.4%, slice thickness of 3.27 mm, slice interval of 3.75 mm, pixel size of 3.64 mm, matrix size of 192 × 192 and scan field-of-view of 70 cm. The PET data were reconstructed in terms of the point spread function (PSF) together with TOF technology.

### Estimated LBM from the LC images: method development and validation

Fat tissues were defined as voxels identified and measured by CT as having CT numbers between −190 and −30 Hounsfield units. A built-in software package of the Advantage Workstation (GE Healthcare) was used to calculate fat volumes (FV). A fat-tissue average density of 0.923g/mL was applied to convert whole-body FV to whole-body fat mass (FM_WB_) [15]. LC images were obtained by truncating whole-body images. The LC region was defined from top level of the thorax to distal point of the ischium. All image slices outside of this region were omitted. The volumes of LC fat tissues (FV_LC_) were measured using the same method.

Traditionally, LBM was equivalent to the fat free mass (FFM) [13]. Because, in practice, the FM_WB_ cannot be measured in conventional PET/CT examination, the relationships between FM_WB_ and FV_LC_ were analysed in the populations from the equation-development group, and an equation was developed using FM_WB_ as the dependent variable and FV_LC_ as independent variables. Subsequently, the LBM could be estimated from LC images (LBM_LC_) according to the following equation:

1 $$ {\mathrm{LBM}}_{\mathrm{LC}}\left(\mathrm{kg}\right)=\mathrm{W}\ \left(\mathrm{kg}\right)-\left(\alpha +\beta \times {\mathrm{FV}}_{\mathrm{LC}}\right)\ \left(\mathrm{kg}\right) $$

where *α* and *β* are the intercept and slope of the equation we developed, respectively.

To test the reliability of this method, LBM by LC images was compared with those derived by the James PE in an independent sample of 97 patients from the validation group. The reference standard was the measurement of LBM from whole-body CT. The James PE was defined as follows:

$$ {\mathrm{LBM}}_{\mathrm{PE}}\left(\mathrm{kg}\right)=1.10\times \mathrm{W}-120\times {\left(\mathrm{W}/\mathrm{H}\right)}^2\left(\mathrm{for}\ \mathrm{male}\right) $$

2 $$ {\mathrm{LBM}}_{\mathrm{PE}}\left(\mathrm{kg}\right)=1.07\times \mathrm{W}-148\times {\left(\mathrm{W}/\mathrm{H}\right)}^2\left(\mathrm{for}\ \mathrm{female}\right) $$

where *W* is weight in kilograms, and *H* is height in centimeters.

### PERCIST evaluation

The therapeutic responses were analysed with PET volume computer-assisted reading (PET VCAR) of the Advantage Workstation (GE Healthcare). PET VCAR, using the James PE to estimate LBM and then measuring the peak SUL (SUL_peak_) of target lesion (SUL_peak-PE_), is one program the clinician can use to assist in monitoring treatment response according to PERCIST 1.0. This software can use an iterative adaptive segmentation algorithm to find a threshold value that separated the target volume from the background tissue by weighting the maximum SUV (SUV_max_) and mean SUV (SUV_mean_) within the target volume with a default weighting factor of 0.5. A volume of interest (VOI) is placed over the tumor, and then, the software automatically measures the SUL_peak-PE_ within the entire tumor. Adjustment of the estimated tumor surface was sometimes needed to include the entire tumor within the margins of the volume of interest. The detailed instructions for PET VCAR were described in our previous study [16]. According to the LBM_PE_ calculated by Eq. 2 and the SUL_peak-PE_ of target lesion measured by PET VCAR, the LC image-based SUL_peak_ (SUL_peak-LC_) of the same target lesion could be calculated.

As defined in PERCIST 1.0 [5], the normal background region (a 3-cm diameter spherical region of interest [ROI]) was automatically delineated by PET VCAR in the right lobe of the liver. SUL_peak_ of baseline lesion at least 1.5-fold greater than liver SUL_mean_ + 2 SDs. If the liver is abnormal, primary tumour should have uptake > 2.0 × SUL_mean_ of blood pool in 1-cm-diameter ROI in descending thoracic aorta extended over 2-cm *z*-axis. Measurable lesion sizes should be 2 cm or larger in diameter for accurate measurement, though smaller lesions of sufficient ^18^F-FDG uptake, including those not well seen anatomically, can be assessed. We chose the hottest lesion as the target lesion on the baseline and subsequent follow-up scans. The hottest lesion on the follow-up scan could be a lesion different from the previously measured lesion, on the assumption that it had been present since baseline. On the basis of the variation of SUL_peak-PE_ and SUL_peak-LC_ between the baseline and follow-up scans, patients were classified as partial metabolic response (PMR), stable metabolic disease (SMD), and progressive metabolic disease (PMD) separately according to PERCIST 1.0.

### Statistical analysis

The patients’ characteristics were presented as mean values and standard deviations. The unpaired *t* test was used to analyse the differences in patients’ characteristics between the equation-development and validation groups. For the equation-development group, Pearson correlation coefficients (*r*) were used to evaluate the relationship between the FV_LC_ and FM_WB_. Then, simple linear regression was applied to generate equation to estimate FM_WB_ from FV_LC_. The populations from the validation group were used for cross-validation: paired *t* test and Bland-Altman plots were used separately to examine the difference and agreement between the outcomes of LBM_PE_ and LBM_LC_ and the reference standard.

The paired *t* test was used to evaluate the differences in the same parameters between before and after treatment for the application group. Concordance and differences among the PERCIST 1.0 results of these two methods were assessed using Cohen’s κ coefficient and Wilcoxon’s signed-ranks test. Furthermore, to evaluate the impact of the LBM algorithm on assessment of the therapeutic response, we first analysed the distribution of SUL_peak-PE_ variation in patients with discordant response classifications. Subsequently, the variation of the LBM_PE_ and LBM_LC_ was evaluated in these patients. Graphs and analyses were performed using Prism GraphPad and the SPSS software.

## Results

### Patient characteristics

Patients’ clinical characteristics and parameters in the equation-development, validation and application groups are shown in Table 1. The patients’ characteristics of the equation-development group were not significantly different from those of the validation group (*P*>0.05). For the application group, there were no significant differences in blood glucose levels, injected doses of ^18^F-FDG, and uptake times between baseline and follow-up examinations (*P*>0.05). The interval between both PET/CT examinations was 136±51 days.

**Table 1:** Clinical characteristics and parameters of the patients in the equation-development, validation and application groups

Parameter	Equation-development group		Validation group		Application group	
	Men (*n*=106) Mean ± SD	Women (*n*=93) Mean ± SD	Men (*n*=54) Mean ± SD	Women (*n*=43) Mean ± SD	Before treatment Mean ± SD	After treatment Mean ± SD
Age (year)	53.5 ± 16.7	52.1 ± 14.8	54.0 ± 15.3	53.0 ± 11.6	54.5 ± 14.9	54.5 ± 14.9
Height (cm)	166.6 ± 6.7	156.3 ± 5.9	168.3 ± 6.5	156.6 ± 5.9	167.6 ± 5.6	167.6 ± 5.6
Weight (kg)	63.5 ± 15.1	56.2 ± 9.2	63.6 ± 10.4	55.7 ± 9.5	63.3 ± 9.6	59.6 ± 10.1
BMI (kg/m^2^)	22.5 ± 3.5	23.0 ± 3.4	22.3 ± 3.2	22.7 ± 3.9	22.4 ± 3.3	21.8 ± 3.5
Blood glucose level (mg/dL)	—	—	—	—	102.6 ± 15.6	106.2 ± 13.6
Dose of ^18^F-FDG (mCi)	—	—	—	—	6.7±0.7	6.5 ± 0.7
Uptake time (min)	—	—	—	—	60.5±4.3	60.4 ± 4.5

### Patient-specific LBM equation and cross-validation

For patients from the equation-development group, the mean FV_LC_ were 11084.01 cm^3^ (range of 1362.69–31516.25 cm^3^) and the mean FM_WB_ was 17.71 kg (range of 2.55–45.48 kg). There was a strong correlation between them (*r*=0.977, *P*<0.0001). According to this relationship, a patient-specific equation for LBM from FV_LC_ was established:

3 $$ {\mathrm{LBM}}_{\mathrm{LC}}\left(\mathrm{kg}\right)=\mathrm{W}\ \left(\mathrm{kg}\right)-\left(2.892+1.3337\times {\mathrm{FV}}_{\mathrm{LC}}\right)\ \left(\mathrm{kg}\right) $$

**Fig. 1:**
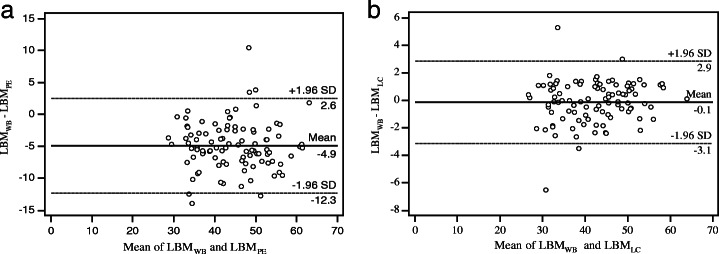
Bland-Altman plots of LBM computed using the James PE (**a**) and the method we developed (**b**) with LBM_WB_ as reference standard

For patients from the validation group, the results of LBM_PE_, LBM_LC_ and LBM_WB_ were 47.01 ± 8.38, 42.26 ± 8.23, and 42.13 ± 8.43 kg, respectively. The LBM_PE_ results were significantly higher than the LBM_WB_ results (*P*<0.001), according to the paired *t* test, whereas the difference was not statistically significant between the LBM_LC_ and LBM_WB_ (*P*=0.408). Bland–Altman plots for each of estimated LBMs are shown in Fig. 1. Compared with LBM_PE_, LBM_LC_ exhibited stronger agreement with LBM_WB_, with closer mean (−0.1 kg) to LBM_WB_ mean and narrower 95 % CI (−3.1; +2.9 kg).

### Comparison of treatment response assessments between PE-based and LC images-based PERCIST

With PE-based PERCIST 1.0, the percentage change in SUL_peak_ was –39.78% ± 18.52 and +34.03% ± 19.18, respectively, in the groups of tumours showing a decrease and an increase in ^18^F-FDG uptake, with 47 patients showing PMD, 82 SMD and 112 PMR. With LC image-based PERCIST 1.0, the percentage change in SUL_peak_ was –39.07% ± 17.88 and +34.19% ± 18.85, respectively, in the groups of tumours showing a decrease and an increase in ^18^F-FDG uptake, with 53 patients showing PMD, 75 SMD and 113 PMR. PE-based and LC image-based PERCIST 1.0 classifications were discordant in 27 patients (11.2 %), with an almost perfect agreement in response classification between the two assessments (κ=0.823, *P*=0.837; Table 2). The reason for the inconsistency in the evaluation results was due to the difference between the change rate of the PE-based SUL_peak_ and the LC images-based exceeding the threshold.

**Table 2:** Comparison of treatment response assessments between PE-based and LC images-based PERCIST

PE-based	LC images-based			
	PMD	SMD	PMR	Total
PMD	41	6	0	47
SMD	12	65	5	82
PMR	0	4	108	112
Total	53	75	113	241

*κ* = 0.823, indicating perfect agreement between the two assessments

*P* = 0.837, Wilcoxon’s signed-rank test, indicating no significant differences between the two assessments

### Distribution of the percentage change of SUL_peak_ in patients with discordant classifications

The distributions of percentage change in SUL_peak-PE_ for patients with discordant response classifications were all within the interval above or below 10% from the threshold (± 30%). Over 10 %, although the variations of SUL_peak-LC_ were different with SUL_peak-PE_, these differences were not enough to change the original classifications. For all patients from the application group, a total of 62 patients’ percentage change of SUL_peak-PE_ distributed in this interval.

**Fig. 2:**
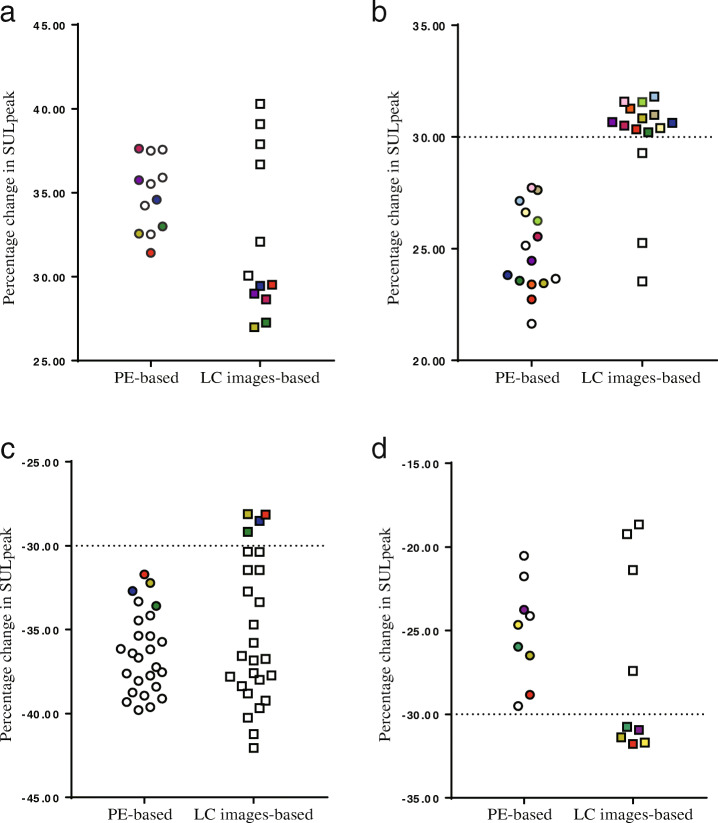
The distribution of percentage change of PE-based SUL_peak_ and corresponding LC images-based SUL_peak_ in 62 patients. Dotted lines illustrate the thresholds of PERCIST 1.0 requirement. Color icons illustrate the patients with discordant response classification. **a** Six patients classified as PMD by PE-based PERCIST 1.0 were reclassified as SMD by LC images-based PERCIST 1.0; **b** 12 patients classified as SMD by PE-based PERCIST 1.0 were reclassified as PMD by LC images-based PERCIST 1.0; **c** 4 patients classified as PMR by PE-based PERCIST 1.0 were reclassified as SMD by LC images-based PERCIST 1.0; **d** 5 patients classified as SMD by PE-based PERCIST 1.0 were reclassified as PMR by LC images-based PERCIST 1.0

The percentage change of 62 patients’ SUL_peak-PE_ and SUL_peak-LC_ are shown in Fig. 2. Using 5% interval as minimum unit, a total of 24 patients’ percentage change of SUL_peak-PE_ were found in the interval above or below 5% from the threshold, and 17 (70.8 %) discordances were found in this region. In addition, 38 patients’ PE-based SUL_peak_ were found in the interval above or below 5–10% from the threshold, and 10 (26.3%) discordances were found in this region.

### Impact of LBM-dependent variation on PERCIST evaluation

Of 27 patients with discordant response classifications, 16 patients’ LBM showed a decrease at the follow-up examination, leading to PMR being changed to SMD and SMD to PMD. The reduction percentages of LBM_PE_ (7.2% ± 2.2) were significantly higher than those of LBM_LC_ (2.8% ± 1.8) (*P*<0.001). Five patients’ LBM showed increase, resulting in changes from SMD to PMR, and PMD to SMD. The increase percentages of LBM_PE_ (5.7% ± 1.5) were significantly higher than those of LBM_LC_ (1.8% ± 1.7) (*P*=0.001). Furthermore, six patients’ weight increase and therefore LBM_PE_ showed increase (1.8% ± 1.0) at the follow-up examination, whereas LBM_LC_ showed decrease (4.8% ± 1.9), resulting in changes from SMD to PMR, and PMD to SMD.

## Discussion

In this study, we proposed a novel and reliable method to estimate the patient’s whole-body LBM from the LC images, and the results of this method showed an excellent agreement with the LBM obtained from the whole-body CT. Furthermore, and more importantly, we found that the classifications of PERCIST 1.0 according to the SUL_peak-PE_ could be reclassified according to the SUL_peak-LC_. The closer the percentage changes of SUL_peak_ to the threshold, the greater the change that could have happened.

CT technology is considered one of the preferred approaches for measuring FM [10], but the application of this technology is limited by the radiation exposure. The previous studies demonstrated that the single abdominal image was highly correlated with the total volume of fat tissue (*r*=0.88–0.963) [17–19]. Accordingly, some investigators proposed, as a compromise between accuracy and reduced radiation burden, using a single cross-sectional image at the abdomen to predict whole-body FM [19, 20]. In this study, we analysed the correlation between a wider range of LC fat volumes and whole-body FM. Similarly, the results showed that the FV_LC_ were significantly correlated with the FM_WB_ (*r*=0.977). Compared with single-slice, the region of LC contained more fat tissues in the body and theoretically could enable estimation of FM_WB_ more accurately.

Previous studies demonstrated that the LBM from the LC images was more accurate than the results obtained from the James PE. In a study by Chan [12], the LBM was estimated from the James PE and the LC images (at least from skull base to 5 cm below the pelvis). The results showed that the LBM was overestimated by the James PE, but not by the LC images method. The reliability of this method was confirmed in a later study [13]. Similar results were obtained in this study. We found that the LBM_PE_ was significantly higher than the reference standard, but not for the LBM_LC_. The Bland–Altman plots showed that the results of LBM_LC_ were more accurate than those of LBM_PE_.

A reasonable explanation for the heterogeneity of LBM_PE_ is that the LBM has ethnicity specificity [21], the James PE was derived from Caucasian populations and was therefore not suitable to apply to a different ethnic group [22]. On the other hand, one major advantage of the method we proposed over the James PE is that it is based mainly on the directly measured LBM of an individual, albeit incomplete, rather than on an assumed similarity between the subject and some specific study population. The accuracy of this method has been proved by the validation group. Furthermore, different from the method proposed by Chan [12], who estimated LBM by using the relative contribution of FM from a larger LC (from the eye to thigh), the method we proposed in this study concerned using the relationship between FV_LC_ and FM_WB_ to estimate LBM. The reason for selecting the region of the thorax to ischium is that the conventional PET/CT scans definitely included this anatomic region, and the anatomical location is clear and easy to popularise.

Through the clinical application, we found that PE-based and LC image-based PERCIST 1.0 was discordant in 27 (11.2 %) patients. These discordances were due to differences in the values of SUL_peak-PE_ and SUL_peak-LC_ and, more important, to the variance in LBM between the baseline and follow-up scans. In general, patients who received cancer treatment suffered from weight loss, especially the skeletal muscle loss [23, 24]. In this study, 16 of 27 patients had a decrease in LBM after treatment, leading to the classifications of PMR and SMD determined by PE-based PERCIST 1.0 and were reclassified as SMD and PMD by LC image-based PERCIST 1.0. These differences illustrate that the percentage of reduction of LBM_PE_ was significantly higher than that of LBM_LC_ (*P*<0.001), and the percentage change of SUL_peak-PE_ was therefore smaller than that of SUL_peak-LC_. These could lead to worse classifications when the percentage change of SUL_peak-LC_ exceeded the thresholds. In addition, five of 27 patients had an increase in LBM after treatment; conversely, the response evaluation based on LC image-based PERCIST 1.0 tended to be more optimistic, from SMD and PMD reclassified as PMR and SMD.

It is noteworthy that six of 27 patients had an increase in body weight and therefore in LBM_PE_ after treatment, but LBM_LC_ showed a decrease, resulting in more optimistic classifications based on LC images-based PERCIST 1.0. These discrepancies are most likely explained by a state, for patients with chronic diseases, in which the loss of muscle mass was associated with preserved or even increased body fat content [25]. Multiple reasons for this state have been suggested, including received chemotherapy, decreased physical activity, increased total caloric intake, altered endocrine function and inflammation [25, 26]. There could be marked weakness despite normal or even increased weight, causing the James PE derived from a population of normal health to produce a larger error.

Although heterogeneity was found only in the region close to the threshold, it is also crucial, because for individual patients, different response classifications may lead to different treatment programs. To ensure the accuracy of response evaluation, PERCIST 1.0 describes in detail methods for controlling the quality of ^18^F-FDG PET imaging conditions and provides a much more detailed framework for lesion selection, region of interest definition and response classification [5]. However, in this study, we found that on the basis of standardised PET/CT scanning process and quality control, accurate measurement of LBM is also essential to ensure the accuracy of evaluation.

The current study had some limitations. First, only 6 obese patients (BMI ≥ 30 kg/m^2^) included in the equation-development group. Whether this equation is suitable for obese patients needs further verification. Large subject samples with wide ranges of body mass and height are needed in the future study for equation development. Second, we only analyse the James PE which commonly incorporated in the modern PET/CT systems. Other formulas, especially the Janmahasatian PE [27] which have been shown to provide a better LBM estimation than the James PE [9], should be evaluated in future study. Finally, we only found the response classifications by PE-based and LC image-based PERCIST 1.0 were discordant in some patients. Although, theoretically, more accurate measurement of LBM_LC_ should produce more accurate PERCIST 1.0 classifications than LBM_PE_, further prospective study is needed to investigate the correlation of these discordances with pathological examinations to confirm this conclusion.

## Conclusions

The whole-body LBM could be estimated from LC CT data acquired in PET/CT examinations. The patient-specific LBM was more accurate than results obtained by the James PE, which has been routinely used in the modern PET/CT system. LBM algorithm-dependent variability in PERCIST 1.0 classification is a notable issue. SUV normalised by LC images-based LBM could change PERCIST 1.0 response classifications based on LBM estimated by the James PE, especially for patients with a percentage change of SUL_peak_ close to the threshold. The degree of variability is related to changes in LBM before and after treatment.

## Abbreviations

SUV: Standardised uptake value
PERCIST 1.0: PET evaluation response criteria in solid tumours 1.0
LBM: Lean body mass
BMI: Body mass index
PE: Predictive equation
FMWB: Whole-body fat mass
FVLC: Volumes of limited-coverage fat tissues
LBMPE: LBM estimated from James PE
LBMLC: LBM estimated from trunk CT images
SULpeak-PE: SUV corrected for LBMPE
SULpeak-LC: SUV corrected for LBMLC
PMR: Partial metabolic response
SMD: Stable metabolic disease
PMD: Progressive metabolic disease
